# Draft genome sequence of *Bifidobacterium breve* DSM 32583, isolated from human milk

**DOI:** 10.1128/MRA.00412-23

**Published:** 2023-10-10

**Authors:** Magdalena Kujawska, Monika Schaubeck, Lindsay J. Hall, Klaus Neuhaus

**Affiliations:** 1 Chair of Intestinal Microbiome, TUM School of Life Sciences and ZIEL–Institute for Food & Health, Technische Universität München, Weihenstephaner Berg, Freising, Germany; 2 HiPP GmbH & Co. Vertrieb KG, Pfaffenhofen (Ilm), Germany; 3 Gut Microbes and Health, Quadram Institute Bioscience, Norwich Research Park, Norwich, United Kingdom; 4 Norwich Medical School, University of East Anglia, Norwich Research Park, Norwich, United Kingdom; 5 Core Facility Microbiome, ZIEL–Institute for Food & Health, Technische Universität München, Weihenstephaner Berg, Freising, Germany; 6 Weihenstephan Microbial Strain Collection, ZIEL–Institute for Food & Health, Technische Universität München, Weihenstephaner Berg, Freising, Germany; Wellesley College, Wellesley, Massachusetts, USA

**Keywords:** *Bifidobacterium*, genome, sequencing

## Abstract

Here, we describe the draft genome sequence of *Bifidobacterium breve* DSM 32583 isolated from human milk obtained from a healthy mother. Potentially, this *B. breve* strain could serve as a probiotic.

## ANNOUNCEMENT

*Bifidobacterium breve* can be found in the gastrointestinal tract, vagina, and breast milk of humans and other mammals. Its presence has been associated with improved health (1). Particular strains of *B. breve* have been suggested to exhibit probiotic properties (2).

We report the draft genome of *B. breve* DSM 32583, isolated from human milk. Healthy women, after normal full-term pregnancy, without mastitis and other perinatal problems were enrolled as donors. Milk samples, collected 7 days after delivery, were cooled and screened the same day for *Bifidobacterium* spp. Dilutions were prepared anaerobically (N_2_:H_2_:CO_2_, 85:10:5), plated on de Man, Rogosa, and Sharpe agar (Oxoid, UK) supplemented with L-cysteine (0.5 g/L) or on *trans*-galactosylated oligosaccharide agar (Merck, Germany) (3), and grown for 48 h at 37°C. The isolate designated *B. breve* DSM 32583 showed higher acid survival, and its evaluation as a probiotic (4, 5) is ongoing. The isolate was deposited as DSM 32583 and WS 5622 in the German Collection of Microorganisms and Cell Cultures (DSMZ, Braunschweig, Germany) and the Weihenstephan Strain Collection (Freising, Germany), respectively.

To extract genomic DNA, *B. breve* DSM 32583 was grown anaerobically on Trypticase soy agar (Carl Roth, Germany) for 48h at 37°C, after which bacteria were lysed mechanically in FastPrep-24 instrument (MP Biomedicals, USA). DNA was recovered using cetyltrimethyl ammonium bromide (CTAB) (Sigma-Aldrich, USA), followed by phenol-chloroform and chloroform:isoamyl alcohol extraction (Carl Roth, Germany). RNA was removed using RNase A (Sigma-Aldrich, USA) (6) and the cleaned DNA was fragmented in the Covaris sonicator model E220 (Covaris, UK). Libraries were prepared using the TruSeq DNA Kit (Illumina, USA) and sequenced on an Illumina MiSeq using a PE300 v3 cartridge, generating 17,887,510 raw reads with an average read length of 301 bp. Read quality was evaluated with fastqc v0.72 (7), and adapters were removed using Clip v1.0.3 (8). The genome was assembled using Unicycler v0.4.6.0 with default settings (9) implemented in Galaxy (10). Genome completeness was estimated 100% at family level using CheckM v1.0.18 (11).

The genome of *B. breve* DSM 32583 comprises 2,292,381 bp in 11 contigs (*N*_50_ = 657,788 bp), with a G+C content of 58.74%. It shared 98.1% average nucleotide identity over 84.9% sequence coverage with the genome of type strain *B. breve* DSM 20213^T^ (GCA_001025175.1), confirming its affiliation to the *Bifidobacterium breve* taxon (pyANI v0.2.10 using ANIb module) (12, 13). Unless stated otherwise, default parameters were used for all software.

## Data Availability

Raw sequence reads have been deposited at Sequence Read Archive (accession no. SRR24425223). The genome assembly was deposited in GenBank (accession no. JARUHL000000000) and annotated using the NCBI PGAP annotation pipeline v6.5 (14). The version described in this paper is the first version.
